# The Effect of Static Stress on the Anisotropy of Piezoceramics

**DOI:** 10.3390/ma15155186

**Published:** 2022-07-26

**Authors:** V. M. Tsaplev, R. S. Konovalov, S. I. Konovalov

**Affiliations:** Department of Electroacoustics and Ultrasonic Engineering, Saint Petersburg State Electrotechnical University “LETI”, St. Prof. Popov, 5, 197022 Saint Petersburg, Russia; rskonovalov.eut@gmail.com (R.S.K.); sikonovalov.eut@gmail.com (S.I.K.)

**Keywords:** piezoceramics, static compression, stress, anisotropy, elastic moduli, piezomoduli, nonlinearity, higher-order parameters, piezoelectric domains, ultrasonic measurements

## Abstract

The influence of static compressional stress on the anisotropy of piezoelectric ceramics of BaTiO_3_ and PZT types is considered theoretically and experimentally. Static compression changes the domain structure of piezoceramics. These changes occur due to the reorientation of mostly 90° domain axes. As a result, all the parameters of the material change—elastic, piezoelectric, and dielectric. Some of them increase, and some, on the contrary, decrease. Changes occur in a nonlinear way, and higher-order parameters appear. The relationship between the total volume of the reoriented domains and the change in elastic moduli and piezomoduli is theoretically considered. The corresponding theoretical dependences are obtained. To confirm these theoretical dependences, experimental measurements were performed using the ultrasonic pulse-interference method at a frequency of 8 MHz. There is practically no oscillation movement of domain boundaries at this frequency, therefore, the change in the system of elastic and piezoelectric moduli is structural, not dynamic. The possibility of predicting changes in the structure of modules as a result of static compression is shown.

## 1. Introduction

Piezoelectric ceramics [1] are widely used in various MEMS devices [2], electroacoustic devices [3], automation systems [4], electric drives [5,6], and piezoelectric harvesters [7]. In many cases, the piezoelectric elements work under large (and sometimes extreme) mechanical static and/or dynamic loads and in strong or superstrong electric fields [8,9,10]. When working under such conditions, all material parameters mechanical, dielectric, and piezoelectric are subject to change [11]. In addition, we know that piezoceramics are highly nonlinear, and this nonlinearity is inherent in all properties without exception [12]. This means that each zero-order parameter mechanical, piezoelectric, or dielectric also has several higher-order parameters. Thus, the complete set of parameters includes a very large number of components, the simultaneous measurement of which is a difficult and sometimes unsolvable problem. To this, we can add that each parameter also depends on frequency (i.e., it exhibits dispersion), temperature, and time (i.e., it has a strong creep). It was shown earlier [13,14,15] that the relative change in Young’s modulus of perovskite-type piezoceramics (i.e., BaTiO_3_ or PZT with various additives) decreases under the action of uniaxial compression while the frequency increases. Within the frequency range of about 3 MHz, the moduli defect becomes frequency-independent [16]. At the same time, uniaxial compression being applied along different directions changes different elastic moduli in different ways. Some of them increase while others decrease. The same effect appears under a static electric field applied in different directions. This means that uniaxial compression or/and uniaxial electric fields change the degree of anisotropy of piezoceramics. In this paper, we discuss this process, but consider only the action of mechanical load.

## 2. Theory

Piezoelectric ceramics are a homogeneous polycrystalline medium, anisotropic in the polarized state. Homogeneous anisotropic bodies are usually divided into two groups. The first group includes bodies having natural anisotropy, which is a consequence of their lattice structure. The second group includes media that are not single crystals, but have anisotropy of properties determined by artificial or natural orientation of crystalline grains. These are so-called textures [17]. A special place among them is occupied by piezotextures, which include polarized piezoceramics. Piezotextures consist of crystals with piezoelectric properties, but the properties of textures differ significantly from similar properties of their constituent crystals both qualitatively and quantitatively, for example, by the number of piezoelectric constants. This is due to the fact that the distribution of the axes of the crystals that make up the texture leads to an averaging of the values that characterize the piezoelectric effect [18].

Textures can be formed in various ways, for example, by polarization, application of a constant electric field, or mechanical stress of uniaxial compression. The properties of piezoelectric textures depend on the properties of the crystals forming them. These dependences can be obtained in various ways. The approach developed in ref. [19] on the basis of the method proposed earlier in [20] gives very good approximations. This method makes it possible to calculate the change in the degree of anisotropy introduced by the mechanical stress of uniaxial compression.

It is well known [21], the elastic properties of piezoceramics significantly depend on its domain structure. Piezoceramic is a polycrystalline medium with a random angular distribution of the crystallographic axes of individual crystallites. Each crystallite has a polydomain structure. Therefore, apolarized piezoceramic is an isotropic medium. In perovskite-type piezoceramics, such as BaTiO_3_, the crystal lattice within a certain temperature range has a tetragonal structure in which two types of domains are possible: 180° and 90°. Under the influence of external stress or an electric field, the vectors of spontaneous polarization of individual domains tend to reorient themselves in such a way that the internal energy of each individual crystallite would be minimal. The texture obtained as a result of external action depends on the type of this action. Only two directions of polar axis reorientation are possible: 180° and 90°. The external electric field causes mainly 180° reorientations [22], whereas mechanical uniaxial stress leads to 90° rotations of the polar axes. In both cases, piezoceramics become anisotropic. If it is already anisotropic (for example, as a result of polarization), then under the influence of a mechanical uniaxial stress, the degree of anisotropy changes. It can decrease or increase, depending on the mutual orientation of the compression axis and the polar axis.

Polarized piezoceramics, for example, BaTiO_3_ or PZT types, are a type of transversely isotropic media with an axis of symmetry “*Z*” of infinite order. As for piezoelectric properties, ceramics belong to a point-symmetry group ∞•m, whereas the anisotropy of elastic properties is characterized by a symmetry group m•∞:m. These properties can be expressed as matrices [23,24,25]:
*Matrix of piezoelectric moduli:**Matrix of relative permittivity:*$\left[\begin{array}{cccccc} 0 & 0 & 0 & 0 & h_{15} & 0\\ 0 & 0 & 0 & h_{15} & 0 & 0\\ h_{31} & h_{31} & h_{33} & 0 & 0 & 0 \end{array}\right]$$\left[\begin{array}{ccc} \varepsilon_{11} & 0 & 0\\ 0 & \varepsilon_{11} & 0\\ 0 & 0 & \varepsilon_{33} \end{array}\right]$*Matrix of elastic moduli:**Flexibility Matrix:*$\left[\begin{array}{cccccc} c_{11} & c_{12} & c_{13} & 0 & 0 & 0\\ c_{12} & c_{11} & c_{13} & 0 & 0 & 0\\ c_{13} & c_{13} & c_{33} & 0 & 0 & 0\\ 0 & 0 & 0 & c_{44} & 0 & 0\\ 0 & 0 & 0 & 0 & c_{44} & 0\\ 0 & 0 & 0 & 0 & 0 & c_{66}=\frac{c_{11}-c_{12}}{2} \end{array}\right]$$\left[\begin{array}{cccccc} s_{11} & s_{12} & s_{13} & 0 & 0 & 0\\ s_{12} & s_{11} & s_{13} & 0 & 0 & 0\\ s_{13} & s_{13} & s_{33} & 0 & 0 & 0\\ 0 & 0 & 0 & s_{44} & 0 & 0\\ 0 & 0 & 0 & 0 & s_{44} & 0\\ 0 & 0 & 0 & 0 & 0 & s_{66}=2\left(s_{11}-s_{12}\right) \end{array}\right]$

The relationship between the constants of individual crystallites and the piezoceramic constants is described by the following system of equations:

(1) $$\left.\begin{array}{c} u_{ij}=s_{ijkl}^{E}\sigma_{kl}+d_{m,ij}E_{m}\\ D_{n}=\varepsilon_{mn}^{\sigma }E_{m}+d_{n,kl}\sigma_{kl} \end{array}\right\}$$

where $u_{ij}$ are components of the elastic strain tensor; $\sigma_{kl}$ components of the elastic stress tensor; $D_{n},E_{m}$ components of electric induction vectors and field strength, respectively; $s_{ijkl}^{E}$ components of the flexibility tensor at a constant field; $d_{mij}$ components of the piezomodulus tensor; and $\varepsilon_{mn}^{\sigma }$ components of the dielectric permittivity tensor.

A pair of Equations (1) is applicable for both a single crystal and piezoceramics in general. Let $u_{ij}^{1}\left(\vartheta ,\varphi ,\psi \right)$ be the components of the strain tensor in a single crystal whose main axes are connected to the coordinate axes of ceramics by Euler angles ϑ,φ,ψ.

The strain of ceramics is determined by the following tensor:

(2) $$u_{ij}=\frac{∭u_{ij}^{1}\left(\vartheta ,\varphi ,\psi \right)f\left(\vartheta ,\varphi ,\psi \right)\sin\vartheta d\vartheta d\varphi d\psi }{∭\sin\vartheta d\vartheta d\varphi d\psi }$$

where f(ϑ,φ,ψ) is angle ϑ,φ,ψ of distribution function $u_{ij}^{1}$.

If ceramics are apolar, single crystals are oriented randomly, so f(ϑ,φ,ψ)=1 and Euler angles can take values within the following intervals:

0≤ϑ≤π; 0≤φ≤2π; 0≤ψ≤2π.

If a texture is somehow created in ceramics, then the polar axes are distributed within a certain solid angle Ω. In this case, the limits of integration in expression (2) will be as follows:

$$\Theta_{1}\leq \vartheta \leq \Theta_{2};\;0\leq \varphi \leq 2\pi ;\;0\leq \psi \leq 2\pi ,$$

with $\Theta_{1},\Theta_{2}<\pi$.

The values $\Theta_{1}$ and $\Theta_{2}$ depend on the type of texture being created. For example, if a texture is created by polarization along the *Z* or *Y* axis, then, as shown in [4], $0\leq \vartheta \leq \frac{\pi }{2}$, since polarization is mainly accompanied by 180° reorientations.

A small number of 90° reorientations also take place. If the texture is created by compression along the *X* axis (which in this case becomes the axis of symmetry of infinite order), then with full reorientation $\Theta_{1}=\frac{\pi }{4}$ and $\Theta_{2}=\frac{3\pi }{4}$, since in this case only 90° reorientations are allowed.

The orientation distribution of the polar axes of monocrystals within a certain fixed solid angle Ω should be uniform, since in the process of creating a texture they can rotate only by a certain angle (90°). Therefore

f(ϑ,φ,ψ)=0 if $\vartheta <\Theta_{1}$ or $\vartheta >\Theta_{2}$,

f(ϑ,φ,ψ)=1 if $\Theta_{1}\leq \vartheta \leq \Theta_{2}$.

With this in mind, we get

(3) $$u_{ij}=\frac{1}{4\pi^{2}\left(\cos\Theta_{1}-\cos\Theta_{2}\right)}\int_{0}^{2\pi }\int_{0}^{2\pi }\int_{\Theta_{1}}^{\Theta_{2}}u_{ij}^{1}\left(\vartheta ,\varphi ,\psi \right)\sin\vartheta d\vartheta d\varphi d\psi$$

Then, the first equation of the system (1) is

(4) $$u_{ij}^{1}=s_{ijkl}^{1E}\sigma_{kl}^{1}+d_{m,ij}^{1}E_{m}^{1},$$

where $\sigma_{kl}^{1}$ and $E_{m}^{1}$ are the mechanical stress and electric field strength acting on the monocrystal accordingly. Substituting (4) into (3), one can obtain

(5) $$u_{ij}=\frac{1}{4\pi^{2}\left(\cos\Theta_{1}-\cos\Theta_{2}\right)}\times \left[\int_{0}^{2\pi }\int_{0}^{2\pi }\int_{\Theta_{1}}^{\Theta_{2}}{s_{ijkl}^{1E}\;\sigma }_{kl}^{1}\sin\vartheta d\vartheta d\varphi d\psi +\right]\int_{0}^{2\pi }\int_{0}^{2\pi }\int_{\Theta_{1}}^{\Theta_{2}}{d_{mij}^{1}\;E}_{m}^{1}\sin\vartheta d\vartheta d\varphi d\psi$$

The values $s_{ijkl}^{1E}$ and $d_{mij}^{1}$ can be expressed in terms of the corresponding values $s_{pqrs}^{•E}$ and $d_{npq}^{•}$, reduced to the main axes of the monocrystal:

(6) $$\left.\begin{array}{c} s_{ijkl}^{1E}=\alpha_{ip}\alpha_{jq}\alpha_{kr}\alpha_{ls}s_{pqrs}^{•E}\\ d_{mij}^{1}=\alpha_{mn}\alpha_{ip}\alpha_{jq}d_{npq}^{•} \end{array}\right\}$$

where α are the guiding cosines of the angles between the main axes of the microcrystalline and the axes associated with the ceramic specimen:

$X_{1}$$X_{2}$$X_{3}$$X_{1}^{1}$$\alpha_{11}$$\alpha_{12}$$\alpha_{13}$$X_{2}^{1}$$\alpha_{21}$$\alpha_{22}$$\alpha_{23}$$X_{3}^{1}$$\alpha_{31}$$\alpha_{32}$$\alpha_{33}$

Substituting (6) into (5) and comparing the resulting expression with (1), we obtain

(7) $$s_{ijkl}^{E}=\frac{1}{4\pi^{2}\left(\cos\Theta_{1}-\cos\Theta_{2}\right)}\int_{0}^{2\pi }\int_{0}^{2\pi }\int_{\Theta_{1}}^{\Theta_{2}}\alpha_{ip}\alpha_{jq}\alpha_{kr}\alpha_{ls}s_{pqrs}^{•E}\sin\vartheta d\vartheta d\varphi d\psi$$

(8) $$d_{mij}=\frac{1}{4\pi^{2}\left(\cos\Theta_{1}-\cos\Theta_{2}\right)}\int_{0}^{2\pi }\int_{0}^{2\pi }\int_{\Theta_{1}}^{\Theta_{2}}\alpha_{mn}\alpha_{ip}\alpha_{jq}d_{npq}^{•}\sin\vartheta d\vartheta d\varphi d\psi$$

(9) $$\varepsilon_{mn}^{\sigma }=\frac{1}{4\pi^{2}\left(\cos\Theta_{1}-\cos\Theta_{2}\right)}\int_{0}^{2\pi }\int_{0}^{2\pi }\int_{\Theta_{1}}^{\Theta_{2}}\alpha_{mp}\alpha_{nq}\alpha_{jq}\varepsilon_{mn}^{•\sigma }\sin\vartheta d\vartheta d\varphi d\psi$$

Using expressions (7)–(9), it is possible to obtain expressions for all parameters of piezoelectric ceramics through the parameters of the monocrystals of which it consists. If, for example, we need to obtain relationships for flexibilities, then, after integrating (7) and taking into account the relationships between Euler angles and guide cosines, we obtain expressions for piezoceramic flexibilities, which are generalizations of the expressions obtained in [20].

In this paper, we do not give a complete set of parameters, limiting ourselves only to those that are of interest for practical usage, i.e., the elasticity and flexibility moduli having indices 11 and 33 under constant values of electric field strength or induction. The other parameters can be obtained similarly and do not cause difficulties. The following expressions were obtained for them:

(10) $$\begin{array}{l} s_{33}^{E}=s_{22}^{E}=\frac{1}{{\cos\Theta }_{1}-{\cos\Theta }_{2}}\left\{s_{11}^{E}\left[-0.056\left(\cos^{5}\Theta_{2}-\cos^{5}\Theta_{1}\right)\right.\right.\\ -0.062(\cos^{3}\Theta_{2}-\cos^{3}\Theta_{1})-0.28(\cos\Theta_{2}-\cos\Theta_{1})]\\ -\left({2s}_{12}^{•E}+s_{66}^{•E}\right)\left[0.0125(\cos^{5}\Theta_{2}-\cos^{5}\Theta_{1})+0.01(\cos^{3}\Theta_{2}-\cos^{3}\Theta_{1})\right.\\ \left.+0.047(\cos\Theta_{2}-\cos\Theta_{1})\right]\\ +2\left({2s}_{13}^{•E}+s_{44}^{•E}\right)\left[0.0375(\cos^{5}\Theta_{2}-\cos^{5}\Theta_{1})-0.042(\cos^{3}\Theta_{2}-\cos^{3}\Theta_{1})\right.\\ \left.-0.0625(\cos\Theta_{2}-\cos\Theta_{1})\right]+s_{33}^{•E}\left[{-0.075(\cos}^{5}\Theta_{2}-\cos^{5}\Theta_{1})\right.\\ \left.\left.+{0.25(\cos}^{3}\Theta_{2}-\cos^{3}\Theta_{1})-0.375(\cos\Theta_{2}-\cos\Theta_{1})\right]\right\} \end{array}$$

and

(11) $$\begin{array}{l} s_{11}^{E}=\frac{1}{{\cos\Theta }_{1}-{\cos\Theta }_{2}}\left\{s_{11}^{•E}\left[{0.15(\cos}^{5}\Theta_{2}-\cos^{5}\Theta_{1})+{0.5(\cos}^{3}\Theta_{2}-\cos^{3}\Theta_{1})\right.\right.\\ \left.-{0.75(\cos\Theta }_{2}-{\cos\Theta }_{1})\right]+\left({2s}_{12}^{•E}{+s}_{66}^{•E}\right)\left[{-0.025(\cos}^{5}\Theta_{2}-\cos^{5}\Theta_{1})\right.\\ \left.+{0.083(\cos}^{3}\Theta_{2}-\cos^{3}\Theta_{1})-{0.125(\cos\Theta }_{2}-{\cos\Theta }_{1})\right]\\ +2\left({2s}_{13}^{•E}+s_{44}^{•E}\right)\left[{0.1(\cos}^{5}\Theta_{2}-\cos^{5}\Theta_{1}){-0.167(\cos}^{3}\Theta_{2}-\cos^{3}\Theta_{1})]\right.\\ \left.{-0.2s}_{33}^{•E}(\cos^{5}\Theta_{2}-\cos^{5}\Theta_{1})\right\} \end{array}$$

These expressions remain valid if instead of flexibilities $s_{ij}^{E}$ and $s_{ij}^{•E}$ we substitute the corresponding flexibilities under constant induction *D* ($s_{ij}^{D}$ and $s_{ij}^{•D}$).

If now, instead of (1), we choose another pair of initial equations:

(12) $$\left.\begin{array}{c} \sigma_{ik}=c_{iklm}^{E}u_{lm}-e_{p,ik}^{u}E_{p}\\ D_{k}\;=\varepsilon_{kp}^{u}E_{p}+e_{k,lm}u_{lm} \end{array}\right\}$$

and perform all the above transformations, it turns out that expressions (10) and (11) remain valid, even if, instead of flexibilities, we substitute the corresponding values of the inverse elastic moduli ${\left(c_{ij}^{E}\right)}^{-1}$ and ${\left(c_{ij}^{•E}\right)}^{-1}$.

Similar relationships are obtained for inverse elastic moduli ${\left(c_{ij}^{D}\right)}^{-1}$ and ${\left(c_{ij}^{•D}\right)}^{-1}$. The elastic moduli can be calculated [26] using the formula

(13) $$c_{ij}^{E,D}=\frac{{\left(-1\right)}^{i+j}\Delta_{ij}^{s}}{\Delta^{s}}$$

where $\Delta^{s}$ is a determinant obtained from a matrix of flexibilities, and $\Delta_{ij}^{s}$ the corresponding minor, obtained by crossing out the *i*-th row and the *j*-th column. The flexibility matrix looks like that given above.

The full set of BaTiO_3_ single crystal-flexibility constants was published by Berlincourt and Jaffe [22]. Table 1 shows the values of flexibilities according to [22] and the values of elastic moduli $c_{ij}^{E,D}$ (in SI) calculated by Formula (13).

Using Table 1 and expressions (10) and (11), some estimates can be made. For example, for apolar ceramic BaTiO_3_, we have

$${\Theta_{1}=0,\;\;\;\;\Theta_{2}=\pi ,\;\;\;\;\;s}_{11}^{E}{=s}_{33}^{E}=0.712\times 10^{-11}\;{\mathrm{Pa}}^{–1}.$$

Young’s Modulus

$$Y_{11}=\frac{1}{s_{11}^{E}}=1.40\times 10^{11}\;\mathrm{Pa}.$$

The real value of Young’s modulus according to experimental data is $Y_{11}=1.1\times 10^{11}$ Pa. In the experiment, we used ordinary porous ceramics. If we introduce a correction for porosity, then the real Young’s modulus is equal to $Y_{11}=1.1\times 10^{11}$ Pa, which corresponds well to the experimental data. If we assume that the polar axes of all domains are completely reoriented under the action of compressive stress, then

$$\Theta_{1}=\frac{\pi }{4},\;\;\;\;\Theta_{2}=\frac{3\pi }{4},\;\;\;\;\;\;s_{11}^{E}=0.591\times 10^{-11}{\;\mathrm{Pa}}^{-1},$$

that is, the reverse flexibility ${\left(s_{11}^{E}\right)}^{-1}$ increases by 20.7%, and taking into account porosity by $\frac{\Delta {\left(s_{11}^{E}\right)}^{-1}}{{\left(s_{11}^{E}\right)}^{-1}}=20\%$. In the same way, we can calculate the increase in reverse flexibility ${\left(s_{11}^{D}\right)}^{-1}$, which will be 12.7%, and taking into account porosity, then 12.2%.

If it is necessary to calculate the change in the inverse flexibilities for the case when only part of the domains of the polar axes were reoriented and the rest of them retained the original direction of the polarization, then expression (2) should be integrated, taking into account the spatial distribution function f(ϑ,φ,ψ) of the polar axes of the domains. We must take into account that in completely apolar ceramics, the polar axes are evenly distributed, and the distribution function has the form

(14) f(ϑ,φ,ψ)=1

for any values of angle ϑ. If we load uniaxially the nonpolar ceramics, then the polar axes of a part of the domains will rotate by a certain angle, and the distribution function will look like this:

(15) $$f(\vartheta ,\varphi ,\psi )=\left\{\begin{array}{l} \;a\;\;\;\;\;\mathrm{if}\frac{\pi }{4}\leq \vartheta \leq \frac{3}{4}\pi \;\\ 1-a\;\mathrm{if}\;0\leq \vartheta <\frac{\pi }{4}\;\mathrm{or}\;\;\frac{3\pi }{4}<\vartheta \leq \pi \; \end{array}\right.,$$

where *a*—is the total volume occupied by the rotated domains.

This distribution function must obey the normalization condition

(16) $$\int_{0}^{2\pi }\int_{0}^{2\pi }\int_{0}^{\pi }f(\vartheta ,\varphi ,\psi )\sin\vartheta d\vartheta d\varphi d\psi =\int_{0}^{2\pi }\int_{0}^{2\pi }\int_{0}^{\pi }\sin\vartheta d\vartheta d\varphi d\psi$$

After integration, we obtain a simple equation:

(17) $$a\left(1-\frac{1}{\sqrt{2}}\right)+\frac{b}{\sqrt{2}}=1$$

The values *a* and b=1−a are determined from the condition $\gamma =\frac{1-a}{a}$, where γ is the ratio of the volume of the reoriented domains (1 − *a*) to the volume of the retained domains (0≤γ≤1). If *a* = 1 (i.e., γ = 0), then we have the first limiting case, a completely apolar ceramic, and if γ = 1, then this is a case of total reorientation.

**Figure 1:**
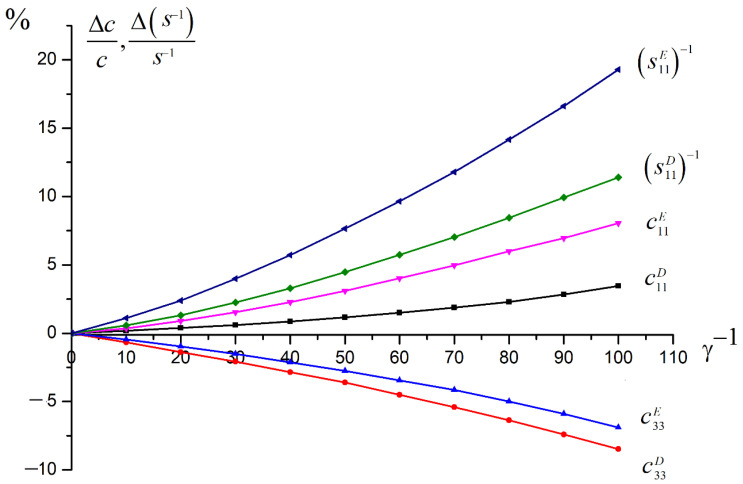
Relative change in the reverse flexibilities ${\left(s_{11}^{E}\right)}^{-1}$ (1) and ${\left(s_{11}^{D}\right)}^{-1}$ (2) and in the elastic moduli $c_{11}^{E}$ (3) and $c_{11}^{D}$ (4) for the apolar piezoceramic BaTiO_3_ depending on the relative volume of the reoriented domains under the compressive stress along the “X” axis. Relative change in the elastic moduli $c_{11}^{E}$ (5) and $c_{11}^{D}$ (6) under the same influence.

The dependences of the elastic constants on γ, taking into account the distribution function (15), are shown in Figure 1.

We can note that the values under constant electrical induction ${\left(s_{11}^{D}\right)}^{-1}$ or $c_{11}^{D}$ change less than the corresponding values change under a constant electric field ${\left(s_{11}^{E}\right)}^{-1}$ or $c_{11}^{E}$. The elastic moduli $c_{11}^{E}$ and $c_{11}^{D}$ change less than corresponding values of reverse flexibilities ${\left(s_{11}^{E}\right)}^{-1}$ and ${\left(s_{11}^{D}\right)}^{-1}$.

## 3. Theoretical Basics of the Experiment

We measured different elastic moduli and their behavior under the action of compressive stresses using the ultrasonic pulse-interference method [19,27]. Here, we consider the possibilities of the high-frequency ultrasound method and the main results obtained.

To describe the propagation of an elastic wave in an anisotropic, in general, medium, additionally deformed by uniaxial compression, we use the approach proposed by Man and Lu [28]. The precompression stress Σ is included as an additional term in the basic elasticity equation:

(18) σ=Σ+c⋅u+H⋅Σ;

where σ—total stress; Σ—pre-compression stress; *u*—elastic strain caused by the elastic wave; *H*—displacement gradient; *c*—stress-dependent modulus of elasticity (fourth-rank tensor).

With small elastic displacements during wave propagation in a prestressed medium, the wave equation has the form

(19) $$\nabla \cdot \sigma =\rho \frac{\partial^{2}p}{{\partial t}^{2}},$$

where ***p***—displacement vector; *ρ*—density.

Using (18), we can write (19) in the tensor form for components

(20) $$\frac{\partial }{{\partial x}_{i}}\left(c_{ijkl}+\sigma_{il}\delta_{jk}\right)\frac{\partial u_{k}}{\partial u_{l}}=\rho {\ddot{p}}_{i},$$

where δ*_jk_*—Kronecker symbol. Stress σ*_il_* may be caused by preload or residual stress. We assume that the material within the width of the ultrasonic beam is homogeneous and the stresses are also homogeneous. The solution of Equation (20) for a plane wave we can write in the form

(21) $$p=p_{0}e^{ik(\mathrm{nr}-vt)},$$

where ***p***_0_—initial (amplitude) displacement vector; *k*—wave number; ***n***—the vector of the unit wave normal in the direction of wave propagation.

Substituting solution (21) into Equation (20) leads to the Christoffel dispersion equation for the case of a stressed anisotropic material

(22) $$\left|c_{ijkl}n_{i}n_{l}+(\sigma_{il}n_{i}n_{l}-\rho v^{2})\delta_{jk}\right|p_{k}=0$$

where *v*—normal (phase) wave propagation velocity; *p_k_*—guiding cosines of the displacement vector in an elastic wave; *n_i_*, *n_l_*—guiding cosines of the wave vector ***n***; δ*_jk_*—Kronecker symbol (unit tensor); *c_ijkl_*—elastic moduli tensor.

Equation (22) was obtained in ref. [29] using the assumption of superplasticity, and was used in refs. [30,31].

Considering the symmetry of the elastic modulus tensor

*c_ijkl_ = c_klij_ = c_jikl_ = c_ijlk_*, (23)

we can replace tensor indexes as follows:

11 → 1     23, 32 → 4

      22 → 2    13, 31 → 5(24)

              33 → 3     12, 21 → 6

Then, the elastic moduli matrix of piezoceramics looks like this:

(25) $$c_{jk}=\left[\begin{array}{cccccc} c_{11} & c_{12} & c_{13} & 0 & 0 & 0\\ c_{21} & c_{22} & c_{23} & 0 & 0 & 0\\ c_{31} & c_{32} & c_{33} & 0 & 0 & 0\\ 0 & 0 & 0 & c_{44} & 0 & 0\\ 0 & 0 & 0 & 0 & c_{55} & 0\\ 0 & 0 & 0 & 0 & 0 & c_{66} \end{array}\right]$$

Then Equation (22) can be written in matrix notation

(26) $$\left|Q_{jk}-\left(\rho v^{2}-\sigma_{il}n_{i}n_{l}\right)\delta_{jk}\right|p_{k}=0,$$

where *Q_jk_*–effective elasticity modulus. For piezoelectric media it can be written as

(27) $$Q_{jk}=c_{ijkl}^{E}\cdot n_{i}n_{l}+\frac{e_{p,ij}\cdot n_{p\cdot n_{j}\cdot }e_{l,nm}\cdot n_{l\cdot n_{m}}}{\varepsilon_{pj}^{u}\cdot n_{p\cdot n_{j}}},$$

with *n_i_, n_l_*—guiding cosines of the wave vector; *e_p,ij_*—piezoconstant tensor; —the relative permittivity tensor.

The first term in (27) defines the elastic moduli realized under a constant electric stress (*E* = const), i.e., in the absence of a piezoelectric reaction of the medium. The second term expresses the piezo addition to elasticity moduli. In this case, elastic moduli are realized under a constant electrical induction (*D* = const), i.e., $Q_{jk}=Q_{ijkl}^{D}$.

The dispersion Equation (22) is a system of three equations with respect to velocity *v*:

(28) $$\left.\begin{array}{l} \left|Q_{11}-\left(\rho v^{2}-\sigma_{il}n_{i}n_{l}\right)\right|p_{1}+Q_{12}p_{2}+Q_{13}p_{3}=0\\ Q_{11}p_{1}+\left|Q_{22}-\left(\rho v^{2}-\sigma_{il}n_{i}n_{l}\right)\right|p_{2}+Q_{23}p_{3}=0\\ Q_{31}p_{1}+Q_{32}p_{3}+\left|Q_{33}-\left(\rho v^{2}-\sigma_{il}n_{i}n_{l}\right)\right|p_{3}=0 \end{array}\right\}.$$

Various combinations of compression directions and the direction of propagation of the ultrasonic wave are possible. In addition, different orientations of the displacement vector are possible during wave propagation, i.e., both longitudinal and shear waves can propagate. In the latter case, the displacement vector can also be oriented at different angles relative to the direction of compression. Various combinations of compression directions, wave-propagation directions, and displacement directions in an elastic wave are also possible with respect to the polarization vector of piezoceramics.

**Figure 2:**
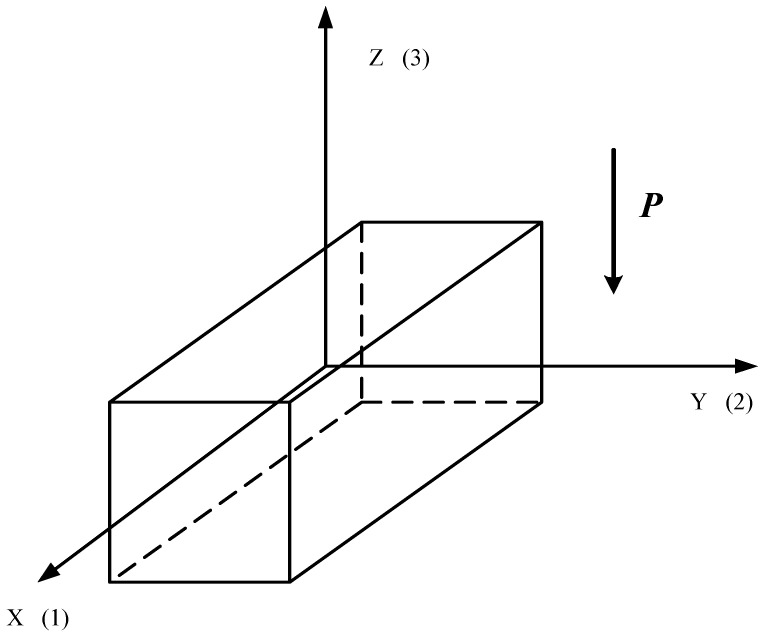
Locations of the axes relative to the polarization vector ***P***.

In Figure 2, the direction of the *Z* axis (3) coincides with the direction of the symmetry axis of infinite order for a transversely isotropic medium, (poled piezoceramics). Locations of the other two coordinate axes—*X* (1) and *Y* (2)—are arbitrary. Vector ***P*** corresponds to the direction of polarization.

**The first case** shows the longitudinal wave, which propagates along the *Z* axis (3). Ceramics is also polarized along the *Z* axis (3). Uniaxial compression is also directed along the *Z* axis (3). The directions of the wave vector and the polarization vector of the wave in this case coincide, and the guiding cosines are the following:

*n*_1_*= n*_2_*=* 0; *n*_3_
*=* 1; *p*_1_
*= p*_2_
*=* 0; *p*_3_
*=* 1.

From the third equation of the system (28) it turns out:

$$Q_{33}-\left(\rho v^{2}-\sigma_{33}\right)=0\mathrm{or}\;c_{3333}^{E}+\frac{e_{3,33}^{2}}{\varepsilon_{33}^{u}}+\sigma_{33}=\rho v_{n}^{2}.$$

And finally:

(29) $$c_{33}^{D}+\sigma_{33}=c_{33}^{E}+\frac{e_{33}^{2}}{\varepsilon_{33}^{u}}+\sigma_{33}=\rho v_{l}^{2},$$

where *v_l_*—the velocity of propagation of an elastic longitudinal wave. Thus, in this case, by measuring the propagation velocity of the elastic wave, it is possible to obtain the elasticity modulus $c_{33}^{D}$.

**In the second case**, the longitudinal wave propagates along the *Y* axis:

*n*_1_ = *n*_3_ = 0; *n*_2_ = 1; *p*_1_ = *p*_3_ = 0; *p*_2_ = 1.

From the second equation of the system (28) we obtain

$$Q_{22}-\left(\rho v^{2}-\sigma_{22}\right)=0\;\mathrm{or}\;c_{2222}^{E}+\frac{e_{2,22}^{2}}{\varepsilon_{22}^{u}}+\sigma_{22}=\rho v_{n}^{2}.$$

Finally:

(30) $$c_{22}^{D}+\sigma_{22}=c_{33}^{E}+\frac{e_{22}^{2}}{\varepsilon_{22}^{u}}+\sigma_{22}=\rho v_{l}^{2},$$

where *v_l_*—the velocity of propagation of an elastic longitudinal wave.

Since

$$c_{22}^{E}=c_{11}^{E}\;,\;\;\;\;\;\;\;\;\;\;\;e_{22}=0,$$

then

(31) $$c_{11}^{E}+\sigma_{22}=\rho v_{l}^{2}.$$

Thus, in this case, by measuring the propagation velocity of the elastic wave, we obtain the elasticity modulus $c_{11}^{E}$.

The same is obtained when the longitudinal wave propagates along the *X*-axis.

**The third case** shows the elastic shear wave propagating along the *Z* axis, and the displacement vector is oriented along the *X* axis. Guiding cosines

*n*_1_ = *n*_2_ = 0; *n*_3_ = 1; *p*_1_ = 1; *p*_2_ = *p*_3_ = 0.

The dispersion equation in this case looks like this:

$$c_{3113}^{E}+\frac{e_{1,31}\cdot e_{3,13}}{\varepsilon_{11}^{u}}+\sigma_{33}=\rho v_{n}^{2},$$

or

$$c_{55}^{E}+\frac{e_{15}\cdot e_{35}}{\varepsilon_{11}^{u}}+\sigma_{33}=\rho v_{n}^{2}.$$

Since

$$c_{55}^{E}=c_{44}^{E}\;,\;\;\;\;\;\;\;\;\;\;\;e_{35}=0\;\;\;\;\;\;\;\;\;\left(e_{15}\neq 0\right),$$

then

(32) $$c_{44}^{E}+\sigma_{22}=\rho v_{t}^{2}.$$

We obtain the elasticity modulus $c_{44}^{E}$. In practice, this case is realized by exciting a shear wave in the sample using a *Y*-piezoquartz transducer, which is located in the *XOY* plane of the sample in such a way that its (i.e., the transducer) optical axis is oriented along the *Y* axis.

**The fourth case.** The elastic shear wave propagates along the *Y* axis and the displacement vector is oriented along the *X* axis. Guiding cosines:

*n*_1_*= n*_3_*=* 0; *n*_2_
*=* 1; *p*_1_
*=* 1; *p*_2_
*= p*_3_
*=* 0.

The dispersion equation in this case looks like the following:

$$c_{2112}^{E}+\frac{e_{1,21}\cdot e_{2,12}}{\varepsilon_{11}^{u}}+\sigma_{22}=\rho v_{n}^{2}\;\mathrm{or}\;c_{66}^{E}+\frac{e_{16}\cdot e_{26}}{\varepsilon_{11}^{u}}+\sigma_{22}=\rho v_{n}^{2}$$

Since, as in the previous case

$$e_{16}=e_{26}=0,$$

Then

(33) $$c_{66}^{E}+\sigma_{22}=\rho v_{t}^{2}.$$

We obtain the elasticity modulus $c_{66}^{E}$. In practice, this case is realized by exciting a shear wave in the sample using an *Y*-piezoquartz transducer, which is located in the *XOZ* plane of the sample in such a way that its (transducer) optical axis is directed along the *X* axis.

The same $c_{66}^{E}$ modulus we can obtain by measuring the velocity of a shear wave propagating along the *X* axis. The piezoquartz *Y*-plate transducer must be located in the *YOZ* plane of the sample in such a way that its (transducer’s) optical axis is directed along the *Y* axis.

Modulus $c_{12}^{E}$ we can calculate using the expression

$$c_{66}=\frac{c_{11}-c_{12}}{2}.$$

**In the fifth case,** the elastic shear wave propagates along the *Y* axis, the displacement vector is oriented along the Z axis. Guiding cosines:

*n*_1_*= n*_3_*=* 0; *n*_2_
*=* 1; *p*_1_
*= p*_2_
*=* 0; *p*_3_ = 1;

The dispersion equation in this case looks like the following:

(34) $$c_{2332}^{E}+\sigma_{22}=\rho v_{t}^{2}\;\mathrm{or}\;c_{44}^{E}+\sigma_{22}=\rho v_{t}^{2}.$$

We obtain the elasticity modulus $c_{44}^{E}$. This is realized by exciting a shear wave in the sample with the help of a *Y*-piezoquartz plate transducer, which is located in the *XOZ* plane of the sample in such a way that its optical axis is directed along the *Z* axis.

The same elasticity modulus $c_{44}^{E}$ can be obtained by measuring the velocity of a shear wave propagating along the *X*-axis. The quartz transducer of the *Y*-slice is located in the *YOZ* plane of the sample in such a way that its (transducer’s) optical axis is directed along the *Z* axis.

## 4. Experimental Setup

Soon after the first practical usages of piezoceramics (mainly in hydroacoustic devices in the 1950s), it turned out that their properties strongly depend on external conditions. This was first established by Bogoroditsky [32]. Mason [20,22,25] was first to begin the intensive studies of these dependences. These first studies were followed by the avalanche-like growth of research in many countries. It is not possible to list all the authors in this work.

A very short list can be found in [15,33], though it is not our main goal. It turned out [34,35,36,37] that the properties of piezoceramics are highly nonlinear, and this nonlinearity, in turn, depends on the frequency. Our more recent studies [13,14] have shown that the frequency dependence of nonlinear properties is mainly determined by the dynamics of oscillations of 90° domain boundaries.

Within the frequency range of about some tens of kHz, the nonlinear acoustoelastic effect is quite strong—the elastic moduli defect can reach 30% or even more [14]. While the frequency increases, defects in elastic moduli, piezomoduli, and dielectric permittivity decrease. Above 3–5 kHz, the elastic moduli become almost completely independent from the frequency, and nonlinear properties are determined mainly by textural changes in the domain structure, i.e., elastic nonlinear dispersion is manifested. This means that the experimental study of changes of the piezoceramic anisotropy must be provided by high-frequency pulsed ultrasonic methods. Within the high-frequency domain, pulsed or pulse-interference methods based on the propagation of ultrasonic pulses of longitudinal or transverse waves are mainly used.

At frequencies exceeding several megahertz, the defect of elastic moduli does not exceed 1–2% percent when the compression stress reaches 100–150 MPa. In this regard, the accuracy of measurement methods is of great importance. The most suitable method here turned out to be the classical one by Williams and Lamb [38].

Another fairly simple classical method by McSkimin [39] is based on the usage of a single transducer. The effect of superimposing odd reflected pulses on each other is used. In essence, this is also a pulse-interference method, but in this method the transducer operates in a combined mode—both radiator and receiver.

These methods are widely known. Their main disadvantage is the inability to measure the velocity of sound along the direction of compression, because of the presence of the compression device. Measurements are possible only in the direction transverse to compression, which greatly limits the usage of these methods.

To measure the velocity and attenuation of sound in various materials, mainly piezoceramic, under the action of uniaxial compression, we have developed the combined method [34], which implements the pulse-interference Williams–Lamb method and the McSkimin pulse-superposition method.

**Figure 3:**
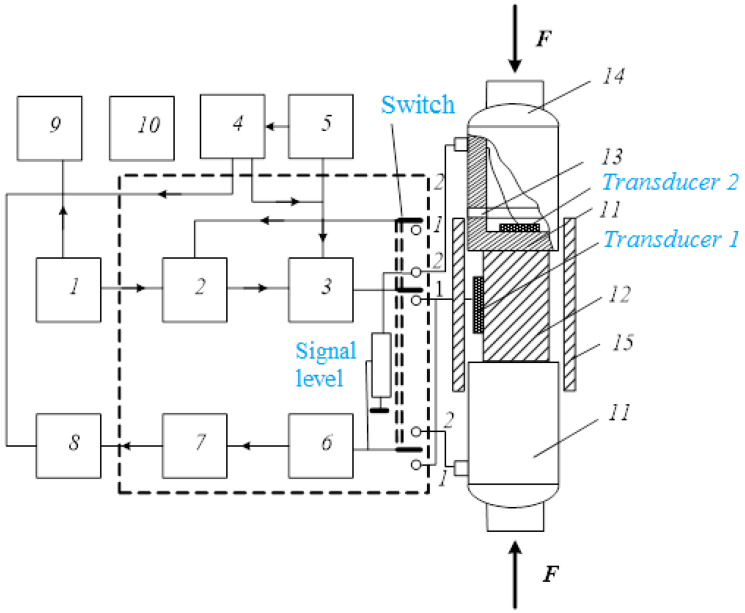
Block diagram of the experimental setup. 1—master oscillator, 2, 3—modulator, 4, 5—pulse oscillators, 6—attenuator, 7—amplifier, 8—oscilloscope, 9—frequency meter, 10—computer, 11—delay lines, 12—specimen, 13—ceramic rings, 14—spherical hinges, 15—thermostatic casing.

This setup (Figure 3) makes it possible to perform experimental measurements of elastic properties of a wide variety of materials under the compressive stress within the selected temperature range, as well as to measure the damping coefficient of elastic waves. The measurements are possible within the frequency range of 3–10 MHz and even more, up to 50–60 MHz.

Measurements are possible by two methods: in the direction transverse to the axis of compression and along the axis of compression.

**In the first case**, the continuous sinusoidal signal from the master oscillator 1 comes to the input of the modulator unit 2, 3, which provides two high-frequency pulse signals from the continuous signal. Their length, as well as the time delay relative to each other, are determined by the operation mode of pulse oscillators 4, 5, providing pulse power to the modulator unit and synchronization of the whole setup.

When measuring the propagation velocity of elastic waves or attenuation in the direction transverse to the axis of compression (when measuring attenuation, the oscillator 5 is switched off), excitatory pulses come to the piezoquartz *transducer* 1 (while the switch is in position 1).

When measuring the propagation velocity or attenuation of elastic waves in the direction transverse to the compression axis (when measuring attenuation, the oscillator 5 is switched off), electric pulses excite the quartz piezoelectric *transducer* 1 (the switch is in position 1). The transducer is glued to the lateral surface of the specimen 12. The received signal, which is a sequence of pulses that have passed through the specimen, comes out from the same *transducer* 1 through a matched calibrated attenuator 6 and amplifier 7 to the input of the oscilloscope 8. The pulse-filling frequency is measured by a frequency meter 9. The pulse-compensation method is generally similar to the Williams–Lamb method.

**In the second case**, when measuring the propagation velocity of elastic waves along the axis of compression, the setup operates in the mode of incomplete modulation of the pulse signal. The operation mode switch is set to position 2. In this variant, the modulator unit generates one pulse signal (oscillator 5 is out) and a sine signal.

The pulse signal comes to the piezoquartz *transducer* 2, which excites an elastic wave. The emitted acoustic pulse goes through two delay lines 11, the specimen 12 and two buffer layers. A continuous signal through the adjusting signal-level device comes directly to the input of the amplifier. Sequential compensations of continuous and pulse signals are recorded by the oscilloscope 8. The latter signal passes the entire acoustic path and is received by a transducer glued to the lower delay line.

The delay lines are flat washers made of hardened tool steel. Their surfaces are treated with a high degree of parallelism, sanded and polished. A piezoelectric *transducer* 2 is glued to the inner plane of each delay line from the piezoquartz X-slice (for excitation and reception of longitudinal waves) or Y-slice (for excitation and reception of shear waves).

Delay lines provide several actions simultaneously.

**First**, it is a power element of the structure. Its purpose is to act by compressive force to the specimen without significant bending deformations. Bending deformations can uncontrollably change the surfaces of contact with *transducer* 2 and contact with the specimen. Longitudinal deformations of the delay lines are feasible, but we can account for them by calculation or by performing control measurements.

**Second**, in the delay lines, as in the specimen, along and back pulses propagate, and there exist several reflected series of pulses—from the upper interface with the specimen, from the lower interface of the specimen—line and from the lower *transducer* 2. It is difficult to share and classify all these reflected pulses; therefore, the thickness of the delay lines should be significantly greater than the length of pulse.

**Third**, when performing temperature measurements, the delay lines must have the same temperature as the specimen, so we must thermally isolate them from the rest of the mechanical system by ceramic rings 13, which take over the temperature difference and are also strong enough to withstand compression. These rings also electrically isolate the steel delay lines from the rest of the structure. This is necessary to perform measurements when the sample is polarized and there are silver electrodes adjacent to the delay lines.

To fulfill all these requirements, the delay lines are made in the form of flat disks made of tool steel. The thickness of the discs is 15 mm, the surfaces must be strictly parallel to each other and carefully treated. The surface to which the piezoquartz transducer is glued must be polished, and the surface of contact with the sample also must be polished. The transducers are glued to the surfaces of the delay lines (by salol (phenyl salicylate, C_6_H_5_O_2_C_6_H_4_OH) for room temperature, or by epoxy resin for measurements under higher temperatures). The layers between the specimen and the delay lines are silicone oil.

Very important elements of the entire structure are spherical hinges 14, the purpose of which is to eliminate possible distortions and nonparallelism between the connected surfaces.

For measurements under varying temperature, the specimen and the delay lines are placed into a thermostatic device (copper casing 15 having thermal insulation) with a heater. The temperature is controlled by a thermocouple with a recording device (not shown in the figure).

The accuracy of the second method is slightly lower than the first one and is 10^–3^ for absolute measurements and 10^−4^ for relative measurements.

Both methods, mutually complementing other, allow us to study the elastic properties of both poled and apolar piezoceramics having different mutual orientations of the compressive force vectors ***F***, the wave vector ***k***, the polarization vector of piezoceramics ***P***, and the displacement vector in the elastic wave, discussed above.

It should be added that measurements on slices of different orientations are also possible, which significantly expands the set of elastic moduli of higher orders available for study.

## 5. Experimental Results

Thus, all the above relations make it possible to experimentally determine the elastic moduli of piezoceramics: $c_{11}^{E}\;,\;{\;c}_{12}^{E},\;\;\;c_{44}^{E},\;\;\;c_{66}^{E}\;$ and also $c_{33}^{D}$ and $c_{44}^{D}$. Based on the results of measurements of the moduli, it is possible to calculate the elastic modulus $c_{12}^{E}$. This makes it possible to experimentally investigate the change in the elastic anisotropy of piezoceramics under the influence of uniaxial compression stress. One can calculate, using the method described above, the change in any modulus of elasticity and obtain dependences similar to those shown in Figure 1.

Commercially available samples of piezoceramics produced by industry were selected for the study. Their properties were determined by the Russian standard [40]. A detailed description of the parameters is available in [41].

**Figure 4:**
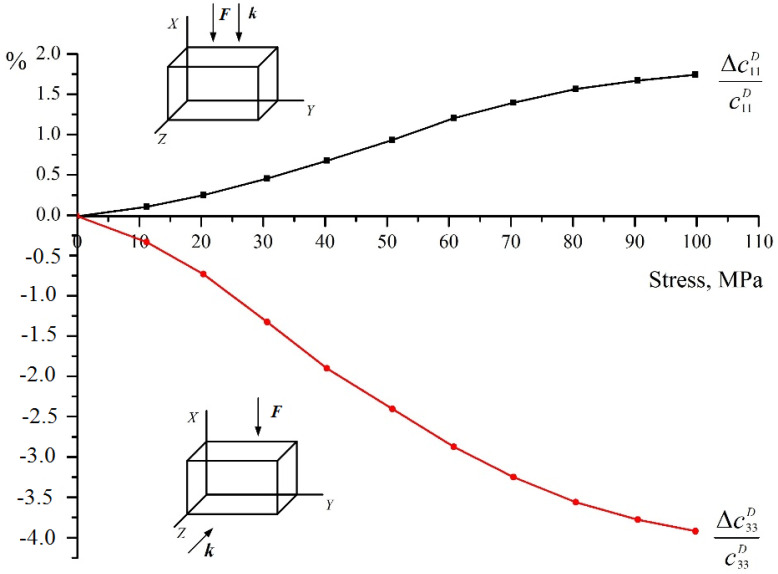
Change of elastic moduli $c_{11}^{D}$ (1) and $c_{33}^{D}$ (2) of BaTiO_3_ piezoceramics under the uniaxial stress along the *X* axis. The arrows show the relative position of the direction of the compression vectors ***F*** and the wave vector ***k***.

The experimental results of the effect of compression stress on the elastic moduli of apolar ceramics of the BaTiO_3_ type are shown in Figure 4 [13].

The compression is directed along the X-axis, which in this case becomes a rotary axis of infinite order. The abscissa axis shows the values of the compression stress σ_11_, and along the ordinate axis is the defect of the module, i.e., its relative change. Curve 1 shows a change in the elastic modulus $c_{11}^{D}$, i.e., that acts along the *X* axis, and curve 2 represents a change in the modulus of elasticity $c_{33}^{D}$ acting perpendicular to the *X* axis (the mutual orientation of the compression vectors ***F*** and the wave vector ***k*** is shown in the figure by arrows).

**Figure 5:**
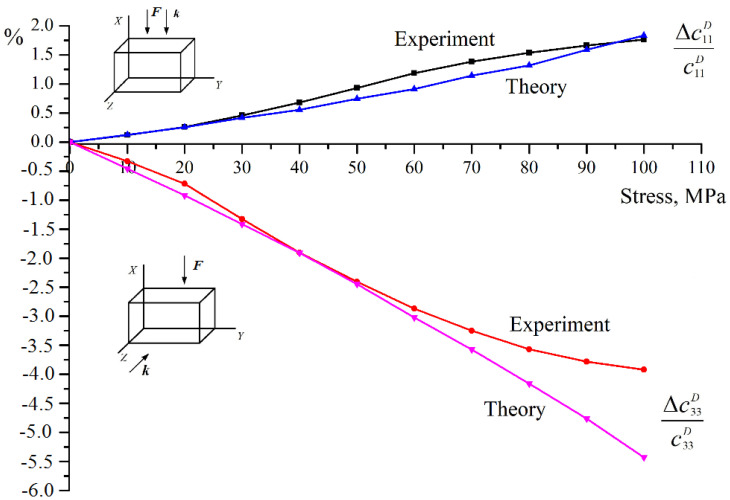
Experimental and computed results for unpoled BaTiO_3_ piezoceramics under the uniaxial stress along the *X* axis.

Figure 5 shows both experimental and computed results for apolar BaTiO_3_ piezoceramics under uniaxial stress along the *X* axis.

Experimental curves were obtained only up to the compression stress values of 110 MPa, due to the limited strength of the samples. This corresponds to the calculated curves shown in the graph in Figure 1 up to the value γ^−1^ = 70, i.e., the total volume of reoriented domains is about 70%. We see a good correspondence with Figure 1. The elastic modulus $c_{11}^{D}$ increases. The maximum change in this modulus is approximately 2%, whereas according to the calculation it is 3.6% (with total domain reorientation). The experimentally measured change in the elastic modulus $c_{33}^{D}$ is about 6% (8% for total domain reorientation), and the calculated increase is 8%. This means that for this type of piezoceramics, the total reorientation of the polar axes of the domains perpendicular to the compression axis is far above this value of compressive stress.

We also studied similar changes in various elastic moduli of poled piezoceramics of different commercial types. The frequency was *f* = 8 MHz with different orientations of the compression vectors ***F***, the wave vector ***k***, and the polarization vector ***p***.

**Figure 6:**
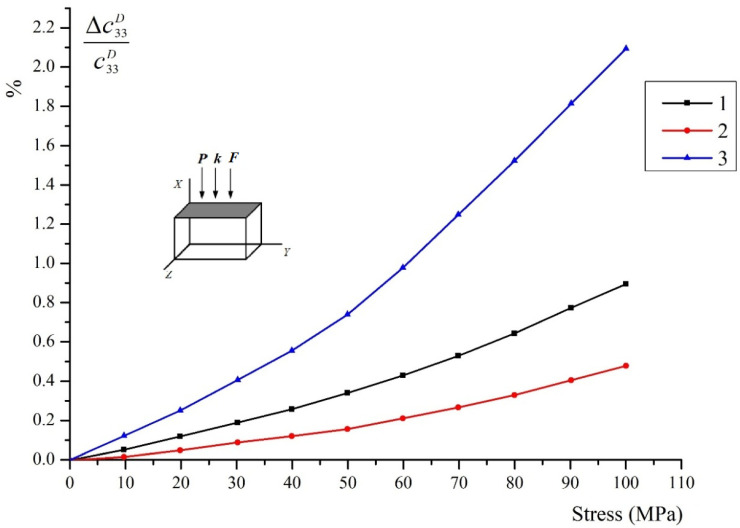
Change in the elastic modulus $c_{33}^{D}$ under uniaxial compression along the Z axis.

Figure 6 shows the dependences of the relative change in the elastic modulus $c_{33}^{D}$ of the following types of piezoceramics:

1. (Ba_0.95_Ca_0.05_)TiO_3_ + 0.75%CoCO_3_
2. Pb_0.95_Sr_0.05_(Zr_0.53_Ti_0.47_)O_3_ + 3%PbO
3. Pb_0.95_Sr_0.05_(Zr_0.53_Ti_0.47_)O_3_ + 1%Nb_2_O_5_.

The mutual orientation of the compression vectors ***F,*** the wave vector ***k***, and the polarization vector of ceramics ***P*** is shown in the figure by arrows. The relative change of the modulus (module defect) as a percentage is shown along the ordinate axis. The “soft” piezoceramic (3) undergoes the greatest change, and the “hard” type (2) undergoes the least. In the latter, the polar domain axes for the most part are still far from total reorientation, since, according the calculations, the limit values for types 2 and 3 should be the same. The change for ceramic 1 (about 1.1% at σ = 100 MPa) is significantly less than the limit determined according to the graph in Figure 1. This is evidently due to two factors.

**Figure 7:**
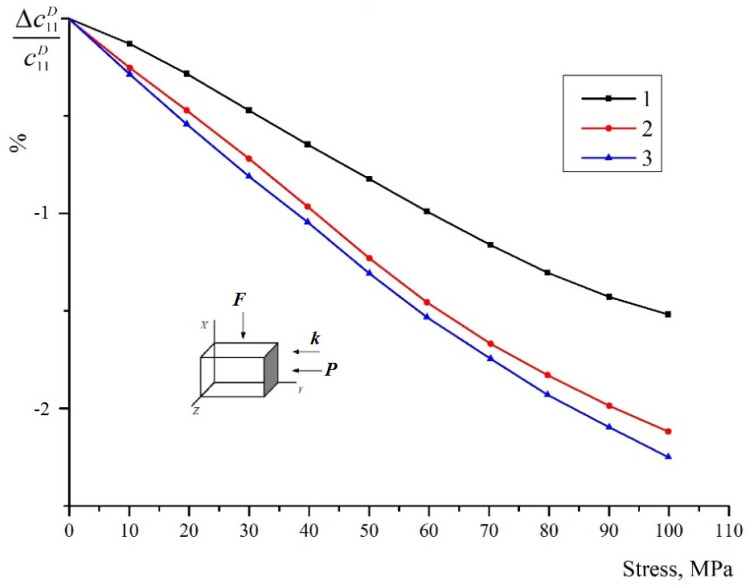
Change in the elastic modulus $c_{11}^{D}$ under the compression across the *Z* axis.

1 is for (Ba_0.95_Ca_0.05_)TiO_3_ + 0.75%CoCO_3_
2 is for Pb_0.95_Sr_0.05_(Zr_0.53_Ti_0.47_)O_3_ + 3%PbO
3 is for Pb_0.95_Sr_0.05_(Zr_0.53_Ti_0.47_)O_3_ + 1%Nb_2_O_5_
1 is for (Ba_0.95_Ca_0.05_)TiO_3_ + 0.75%CoCO_3_
2 is for Pb_0.95_Sr_0.05_(Zr_0.53_Ti_0.47_)O_3_ + 3%PbO
3. is for Pb_0.95_Sr_0.05_(Zr_0.53_Ti_0.47_)O_3_ + 1%Nb_2_O_5_
  (1) After polarization, the axes are distributed not only within the angle ϴ = 90°, but within a slightly larger angle, since the domains for which the angle is close to 90° are not reoriented. Accordingly, the initial value of the elastic modulus $c_{33}^{D}$ will be somewhat larger than for unpoled ceramics.
  (2) Polarization fixes the domain polar axes. After aging, a stable texture is formed and ceramics become “harder”. This is also confirmed by the dependences shown in Figure 7 for the same types of piezoceramics. Compression is now applied not along, but across the polar axis. The convexity of the curves upwards shows that the process of reorientation is close to “saturation”. This is also indicated by the fact that the changes for types No. 2 and No. 3 are practically almost the same.

**Figure 8:**
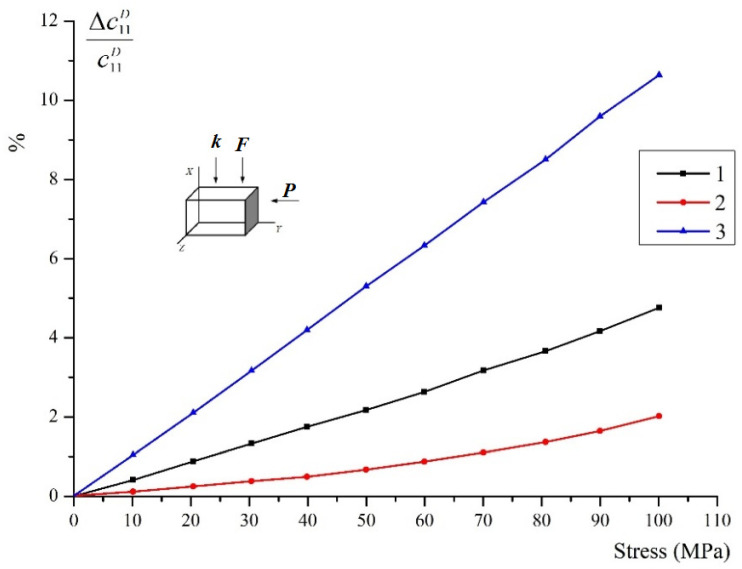
Change in elasticity modulus $c_{11}^{D}$ when compressed across the *Z* axis.

Figure 8 shows the change in the elastic modulus under the compression across the polar axis for the same piezoceramics. It can be noted here that the relative change in the elastic modulus for all types is much more than in the previous case.

1 is for (Ba_0.95_Ca_0.05_)TiO_3_ + 0.75%CoCO_3_

2 is for Pb_0.95_Sr_0.05_(Zr_0.53_Ti_0.47_)O_3_ + 3%PbO

3. is for Pb_0.95_Sr_0.05_(Zr_0.53_Ti_0.47_)O_3_ + 1%Nb_2_O_5_

In general, for the dependences shown in Figure 6, Figure 7 and Figure 8, we can use an angular coefficient $\alpha =\frac{\partial \ln c_{ij}^{E,D}}{\partial \sigma_{kl}}$, where $c_{ij}^{E,D}$ considered an elasticity modulus. The sign of κ depends on the mutual orientation of vectors ***k*** and **σ**, and does not depend on the orientation of vectors ***k*** and ***P***. In those cases, the wave propagates along the compression, i.e., at ***F*** ↓↓ ***k*** coefficient α > 0, and at ***F*** ⊥ ***k*** coefficient α < 0.

The nature of the curve change is associated with a change in the 90° structure of piezoceramics and is determined by a corresponding change in elastic anisotropy in the process of domain reorientation. The creation of a texture quasi-perpendicular to the compression axis causes an increase of rigidity in this direction (α > 0). Accordingly, the elastic moduli measured in the direction parallel to the compression axis increase, and the elastic moduli measured in the perpendicular direction decrease (α < 0).

The presence of the original domain texture in the sample, as can be seen, does not affect the general nature of the dependences under consideration (i.e., the sign of α), since polarization has little effect on the 90° structure. However, the value of α significantly depends on the mutual orientation of the vectors ***k*** and ***P***, and at ***k*** ⊥ ***P***, the value of α is always greater than at ***P*** ↓↓ ***k***. In general, this corresponds to the calculated data, but the value of α largely depends on the fact that polarization and compression create a complex double structure, the calculation of which by the described method is quite possible, but very complex.

## 6. Conclusions

With respect to the results obtained from the previous sections, the following conclusions can be drawn:

1. Different elastic moduli change differently under the influence of compressive mechanical stresses. Some of them increase and some of them decrease, depending on how the directions of compression, wave vector, and polarization of the sample are oriented.
2. Compression leads to change in the anisotropy of piezoceramics. Measuring all major constants and higher-order constants is a very time-consuming task, and is not always possible.
3. The change in various elastic moduli occurs in a nonlinear manner, which indicates the presence of higher-order moduli.
4. The obtained ratios allow us to calculate changes in some parameters based on data obtained as a result of measuring changes in other parameters.
5. In general, the experimental results correspond to the calculated data, but the value of κ largely depends on the fact that polarization and compression create a complex double structure, the calculation of which by the described method is quite possible; however, it is a very difficult problem.

## Figures and Tables

**Table 1:** The full set of BaTiO_3_ single crystal-flexibility constants.

*ij*	$s_{ij}^{E}\times 10^{-11}$	$s_{ij}^{D}\times 10^{-11}$	$c_{ij}^{E}\times 10^{-11}$	$c_{ij}^{D}\times 10^{-11}$
11	0.85	0.725	4.28	2.82
12	–0.235	–0.315	1.79	1.86
13	–0.524	–0.326	1.51	1.412
33	1.57	1.08	1.65	1.78
44	1.84	1.24	0.543	0.807
66	0.884	0.884	1.13	1.13

## Data Availability

Not applicable.
